# Role of phosphoglucomutase in regulating trehalose metabolism in *Nilaparvata lugens*

**DOI:** 10.1007/s13205-020-2053-5

**Published:** 2020-01-23

**Authors:** Bi-Ying Pan, Yong-Kang Liu, Hong-Kai Wu, Xiao-Qing Pang, Shi-Gui Wang, Bin Tang, Cai-Di Xu

**Affiliations:** 1 College of Life and Environmental Sciences, Hangzhou Normal University, Hangzhou, 310036 Zhejiang People’s Republic of China; 2 College of Education, Hangzhou Normal University, Hangzhou, 310036 Zhejiang People’s Republic of China

**Keywords:** *Nilaparvata lugens*, Phosphoglucomutase, Trehalose and glycogen metabolism, Chitin metabolism, RNAi

## Abstract

Phosphoglucomutase (PGM) is a key enzyme in glycolysis and gluconeogenesis, regulating both glycogen and trehalose metabolism in insects. In this study, we explored the potential function of phosphoglucomutase (PGM) using RNA interference technology in *Nilaparvata lugens*, the brown planthopper. *PGM1* and *PGM2* were found highly expressed in the midgut of brown planthoppers, with different expression levels in different instar nymphs. The glycogen, glucose, and trehalose levels were also significantly increased after brown planthoppers were injected with dsRNA targeting PGM1 (dsPGM1) or PGM2 (dsPGM2). In addition, injection of dsPGM1 or dsPGM2 resulted in increased membrane-bound trehalase activity but not soluble trehalase activity. Furthermore, the expression of genes related to trehalose and glycogen metabolism decreased significantly after injection with dsPGM1 and dsPGM2. The expression levels of genes involved in chitin metabolism in the brown planthopper were also significantly decreased and the insects showed wing deformities and difficulty molting following RNAi. We suggest that silencing of PGM1 and PGM2 expression directly inhibits trehalose metabolism, leading to impaired chitin synthesis.

**Electronic supplementary material:**

The online version of this article (10.1007/s13205-020-2053-5) contains supplementary material, which is available to authorized users.

## Introduction

Rice (*Oryza sativa* L.) is the most important cereal crop in China. However, rice is threatened by various pests during production and storage, among which *Nilaparvata lugens* Stål (brown planthopper) is one of the most harmful (Zhang et al. 2017b; Boddupally et al. 2018). *N. lugens* can directly damage rice and spread various rice viral diseases, such as rice grass and rice ragged stunts (Cheng et al. 2013). Most rice production relies on the use of chemical insecticides to control brown planthoppers which inevitably causes environmental pollution. In addition, the use of pesticides also kills the natural predators of brown planthopper and can induce resistance, eventually leading to their resurgence of crop disease (Becker et al. 1996; Tanaka et al. 2000; Nauen and Denholm 2005; Bottrell and Schoenly 2012).

Carbohydrate metabolism is important central because it is a bridge to biosynthetic pathways for proteins, lipids, nucleic acids, and secondary metabolites. Trehalose plays a very important role in the development and physiological activities of insects as an important sugar in haemolymphs (Zhang et al. 2017b; Becker et al. 1996; Elbein et al. 2003; Tang et al. 2010). Glycogen is also another important metabolite (Tolmasky et al. 2001; Liu et al. 2009), which is mainly synthesised and stored in fat bodies. Glycogen can be rapidly converted into trehalose and glucose in other tissues when insects need a supply of energy (Tang et al. 2012). The enzyme phosphoglucomutase (PGM) plays a key role in the interconversion of trehalose and glycogen in insects.

PGM is a conserved enzyme, ubiquitous in animals, plants, and microorganisms catalysing the interconversion of glucose-6-phosphate (G-6-P) and glucose-1-phosphate (G-1-P) (Jin et al. 2018; Egli et al. 2010; Stray-Pedersen et al. 2014; Weyler and Heinzle 2015; Liu 2013). Because glucose-6-phosphate is an important central metabolite, PGM plays an important role in the metabolism of proteins, lipids, and nucleic acids and is key for the development of plants (Egli et al. 2010; Paparelli et al. 2013; Malinova et al. 2014) and some microorganisms (Liu 2013). In humans, there are four PGM isoenzymes namely PGM1, PGM2, PGM3, and PGM5, which are encoded by different genes (Jin et al. 2018).

At present, there are few studies investigating PGMs in insects or invertebrates. Because PGM controls the interconversion between G-6-P and G-1-P, which is the only way to convert trehalose into glycogen, PGM has an important role in trehalose and glycogen metabolism in the brown planthopper. In this study, we further explore the role of PGM in trehalose and glycogen metabolism. A greater understanding of the role of PGM will enable its potential use as a new target for green insecticides.

## Materials and methods

### Experimental materials

*Nilaparvata lugens* was obtained from the Hangzhou population of the China Rice Research Institute. The rice used for feeding (*O. sativa*) was the susceptible Taichung Native 1 (TN1) strain. Brown planthoppers were maintained at a temperature of 25 ± 1 °C, a light dark cycle of 16 h light and 8 h dark, and a relative humidity of 70 ± 5%. The insects used for RNA interference (RNAi) microinjection experiments were nymphs grown to the fifth instar.

### RNA extraction and cDNA preparation

Total RNA was obtained from the brown planthopper by extraction using the TRIzol reagent and the RNA integrity was determined by electrophoresis on 1% agarose gels. The concentration and purity of total RNA was determined using a Nanodrop 2000 spectrophotometer (Thermo Fisher Scientific, Waltham, MA, USA). cDNA was synthesised using the PrimeScript RT reagent Kit with gDNA Eraser (TaKaRa, Nojihigashi, Japan) according to the manufacturer’s instructions.

### Synthesis of dsRNA

The dsRNA primers used for the synthesis of dsPGM1, dsPGM2, and dsGFP were designed using Primer 5 software and are listed in Table 1. cDNA was amplified with the primers of interest as follows: 40 cycles of 95 °C for 30 s, 58 °C for 30 s, and 72 °C for 45 s, followed by a final extension at 72 °C for 10 min. The PCR products were subjected to T cloning and subsequently amplified with primers containing the T7 promoter sequence. To synthesise dsRNA, cross-PCR reactions were performed using the T7 RiboMAX™ Express RNAi System kit (Promega, Madison, WI, USA). The integrity of the dsRNA was determined by gel electrophoresis, and the concentration and purity were measured using NanoDrop 2000.

**Table 1:** Primers used for qRT-PCR and for construction of dsPGM1, dsPGM2, and dsGFP

Primer name	Gene name	Genebank accession number	Forward primer (5′-3′)	Reverse primer (5′-3′)	Length (bp)
QNl18S			CGCTACTACCGATTGAA	GGAAACCTTGTTACGACTT	165
QNlPGM1	Phosphoglucomutase 1	KU556839.1	AACGAGACGGTGGGAGAC	TCCTGGTAAGTGTTGAGCC	127
QNlPGM2	Phosphoglucomutase 2	KU556840.1	AGAGGAAGGTTGGGAGTG	CATAATTCGCGGAGATAAG	141
QNlGP	Glycogen Phosphorylase	KU556838.1	GCTGCCTATGGCTATGGTATTC	TCTGAGTGTTGACCCACTTCTTG	202
QNlGS	Glycogen synthase	KU556837.1	GCTCCAAAGCCTATGTTTCTACTG	TGGTAACCCCTGTCCCTCA	160
QNLUGPase	UDP-Glucose pyrophosphorylase	KU556842.1	ATACAAGATGGCGGCTAA	TTGTGGCAGTTGATAGAGC	136
QNlTPS1	Trehalose-6-phosphate synthase 1	GQ397450	AAGACTGAGGCGAATGGT	AAGGTGGAAATGGAATGTG	154
QNlTPS2	Trehalose-6-phosphate synthase 2	KU556826	AGAGTGGACCGCAACAACA	TCAACGCCGAGAATGACTT	161
QNlTPS3 (Tang et al. 2019)	Trehalose-6-phosphate synthase 3	KU556827	GTGATGCGTCGGTGGCTAT	CCGTTCATCATTGGGCATAGT	224
QNlTRE1-1	Trehalase 1–1	FJ790319	GCCATTGTGGACAGGGTG	CGGTATGAACGAATAGAGCC	132
QNlTRE1-2	Trehalase 1–2	KU556829	GATCGCACGGATGTTTA	AATGGCGTTCAAGTCAA	178
QNlTRE2	Trehalase 2	GQ397451	TCACGGTTGTCCAAGTCT	TGTTTCGTTTCGGCTGT	197
QNlHK	Hexokinase	KU556830	GGTGCGAGAAGAAGTGAAG	GTGAAACCCATTGGTAGAGT	147
QNlGFAT	Glutamine: fructose-6-phosphate aminotransferase	KU556833	CCTCCCAGTTCATCTCGC	CCAAGTTCTTCAAACCCTTTAT	105
NlG6Pase	Glucose-6-phosphatase	KU556841.1	AGACCCTGGCAGTAGAATAG	GGGAAGTGAGCCGAAAT	132
NlG6PI1	Glucose-6-phosphate isomerase 1	KU556832.1	GTTCACGGTCGTCTGGAAAG	TGACTGCTCCGTTTCACTCT	82
QNlG6PI2	Glucose-6-phosphate isomerase 2	KU556831.1	AACAAGGCGACATGGAATCG	ACCATTTGTTCCTGGTTCGC	85
QNlG6PI3	Glucose-6-phosphate isomerase 3	XM_022345379.1	ATGTCACAGTGCATGTCGTG	ACCTGCTCTCATTGATGCCA	120
QNlGNPNA	Glucosamine-6-phosphate *N*-acetyltransferase	KU556834	TGAGCTGCTGAAGACACT	CCTGAATAACGGTGATGTA	179
QNlUAP	UDP-*N*-acetylglucosamine pyrophosphorylase	JF330415	ACGACAGATTAAAGCCGATAC	TACCTTGTCCACCAGCCA	147
QNlCHS1	Chitin synthase 1	AEL88648	CCGCAAACGATTCCTACAGA	AGGTCCTTGACGCTCATTCC	222
QNlCHS1a	Chitin synthase 1a	JQ040014	TGTTCTTGCTACAACTCAATAAA	ACACCAATCCGATAGGCTC	141
QNlCHS1b	Chitin synthase 1b	JQ040013	GCTGTCTTTGCTTTCTTCAT	ACACCAATCCGATAGGCTC	187
ds*NlPGM*1	Phosphoglucomutase 1		GGCAAGCGTTCCTTAGAG	CAGCCACATCCTTTTCATC	553
ds*NlPGM1*-T7	Phosphoglucomutase 1		GGCAAGCGTTCCTTAGAG	CAGCCACATCCTTTTCATC	603
ds*NlPGM2*	Phosphoglucomutase 2		CAACACTTCAAACGGAGGA	CCGCATAGGGACCAGTAA	311
ds*NlPGM2*-T7	Phosphoglucomutase 2		CAACACTTCAAACGGAGGA	CCGCATAGGGACCAGTAA	361
ds*GFP*	Green fluorescent protein		AAGGGCGAGGAGCTGTTCACCG	CAGCAGGACCATGTGATCGCGC	
ds*GFP*-T7	Green fluorescent protein		AAGGGCGAGGAGCTGTTCACCG	CAGCAGGACCATGTGATCGCGC	

T7: GGATCCTAATACGACTCACTATAGG

### Microinjection of the brown planthopper

The brown planthoppers were anesthetised with CO_2_ and then placed in the groove of a pre-prepared agarose gel. Using a standard capillary under a microscope, dsGFP, dsPGM1, and dsPGM2 (50 ng) were injected into the lateral epidermis of the two pairs of hind paws in the brown planthopper chest. The injected brown planthoppers were transferred to a glass tube containing fresh rice, and the surviving brown planthoppers were used for subsequent experiments within 48 h after injection. One hundred brown planthoppers were employed in each treatment and 10 were used in RNA isolation for quantitative real-time PCR (qRT-PCR).

### Experimental materials’ collection

Experimental materials to examine developmental expression patterns in the brown planthopper were collected in triplicate every 24 h, over a period extending from the first day of the fourth instar nymph to the third day of adulthood. In addition, the tissue materials from the head, foot, wing, epidermis, ovary, fat body, and midgut were obtained through anatomical dissection using Leica EZ4 (Leica, Wetzlar, Germany). Different tissue materials were taken from more than 200 adults, and materials for each developmental stage were collected from 10 individuals. All experimental materials were stored at − 80 °C.

### Gene expression by qRT-PCR

Total RNA was extracted from the experimental materials to examine developmental and tissue expression patterns, and cDNA was synthesised as previously described. The relative expression levels of the *PGM1* and *PGM2* genes at the different developmental stages and tissues were analysed by qRT-PCR using the *18S* gene as an internal reference (Zhao et al. 2016). The reaction contained 10 μL of SYBR Premix Ex Taq (TaKaRa, Nojihigashi, Japan), 1 μL of template cDNA, 1 μL of forward primer, 1 μL of reverse primer, in a 20 μL final reaction volume. The sequences of the qRT-PCR primers are shown in Table 1. The PCR amplification procedure included, pre-denaturation at 95 °C for 2 min, 39 cycles of denaturation at 95 °C for 30 s, annealing at 55–60 °C for 30 s, and dissociation curve at 65–95 °C for 5 s.

The relative expression levels of the target genes involved in trehalose and chitin metabolism in the brown planthopper 48 h after RNAi treatment were examined in triplicate using the SYBR Premix Ex Taq kit. The quantitative primers used are shown in Table 1.

### Determination of trehalase activity and carbohydrate content in the brown planthopper after RNAi treatment

Phosphate buffered saline (PBS) was added to the appropriate tissue sample to obtain a final volume of 200 μL, and the tissue was disrupted by grinding and sonication. After disruption, 800 μL of PBS was added, and the sample was centrifuged at 1000*g* for 20 min at 4 °C. The supernatant (350 μL) was removed and centrifuged at 20,800*g* for 1 h at 4 °C. The supernatant was used to measure glucose content, total glycogen content, protein concentration, and soluble trehalase activity, and the pellet was resuspended in PBS to determine glucose content, protein concentration, and membrane-bound trehalase activity. The remaining supernatant was used for determination of total protein and trehalose concentrations. The methods used have been described previously (Zhang et al. 2017a).

### Analysis of NlPGM cDNA and protein sequences

NlPGM1 and NlPGM2 cDNA sequences were compared with other phosphoglucomutase sequences present in GenBank using the BLAST-N and BLAST-X tools available on the National Center for Biotechnology Information (NCBI) website (https://blast.ncbi.nlm.nih.gov/Blast.cgi). Multiple sequence alignment of insect PGMs was performed using the tool available at the multiple sequence alignment website (https://bioinfo.genotoul.fr/multalin/multalin.html) and using DNAMAN software. The neighbour-joining method was used to construct a phylogenetic tree based on the amino acid sequences of known PGMs using MEGA 6.0 software. Bootstrap analysis was carried out and the robustness of each cluster was verified using 1000 replicates. NlPGM protein sequences and other analysis criteria used in this study, including MW, pI, and topology were deduced from the corresponding cDNA sequences using the translation tool on the ExPASy proteome prediction tools website (https://expasy.org/tools/dna.html).

### Statistical analysis

Relative gene copy numbers were determined by qRT-PCR, using the 2^−ΔΔCT^ method (Livaka and Schmittgen 2001). All reactions were performed in triplicate and with three biological replicates. The equation were used as follows (Chen et al. 2018):

$$2^{{ - {\text{DDCT}}}} = 2^{{ - \left[ {\left( {{\text{CT test group}} - {\text{CT test group }}18{\text{S}}} \right) - \left( {{\text{CT control group}} - {\text{CT control }}18{\text{S}}} \right)} \right]}}$$

All the data were analysed using one-way ANOVA and multiple comparisons of the means were conducted using Tukey’s test. Differences were considered statistically significant if the *P* value was between 0.01 and 0.05, and extremely significant if the *P* value was less than 0.01. All data were plotted using SigmaPlot 10.0 software.

## Results

### Analysis of NlPGM1 and NlPGM2 sequences

NlPGM1 (KU556839.1) and NlPGM2 (KU556840.1) cDNAs had open reading frames of 1821 and 1023 bp, respectively. NlPGM1 cDNA encoded a protein of 606 amino acids with a predicted mass of approximately 68.30 kDa and a pI of 5.89 (Fig. S1A). NlPGM2 encoded a protein of 340 amino acids with a predicted mass of approximately 36.82 kDa, and pI of 6.26. (Fig. S1B).

### Phylogenetic and multiple protein alignment analysis

The PGM protein sequences of *Laodelphax striatellus*, *Osmia bicornis bicornis*, *Solenopsis invicta*, *Cimex lectularius*, *Halyomorpha halys*, *Monomorium pharaonis*, and *Ceratina calcarata* were selected to determine the homology by alignment with the NlPGM1 protein sequence. The PGM protein sequences of *L. striatellu*s, *Frankliniella occidentalis*, *Locusta migratoria*, *Melanaphis sacchari*, *Sarracenia flava*, *Acyrthosiphon pisum*, and *Myzus persicae* were selected to determine the homology by alignment with the NlPGM2 protein sequence. The results showed that the insect PGMs were highly conserved: the homology of the PGM1 protein of brown planthopper and other insects ranged from 56.77 to 90.59% (Fig. S2A); the homology of the PGM2 protein ranged from 62.26 to 89.66% (Fig. S2B).

Phylogenetic analysis using MEGA 6.0 software showed that the NlPGM1 and NlPGM2 proteins could be easily distinguished (Fig. S3). In addition, we found that the PGMs of *N. lugens* and *L. striatellus* could be assigned to the same subgroup (Fig. S3).

### Developmental and tissue expression patterns of PGM1 and PGM2

**Fig. 1:**
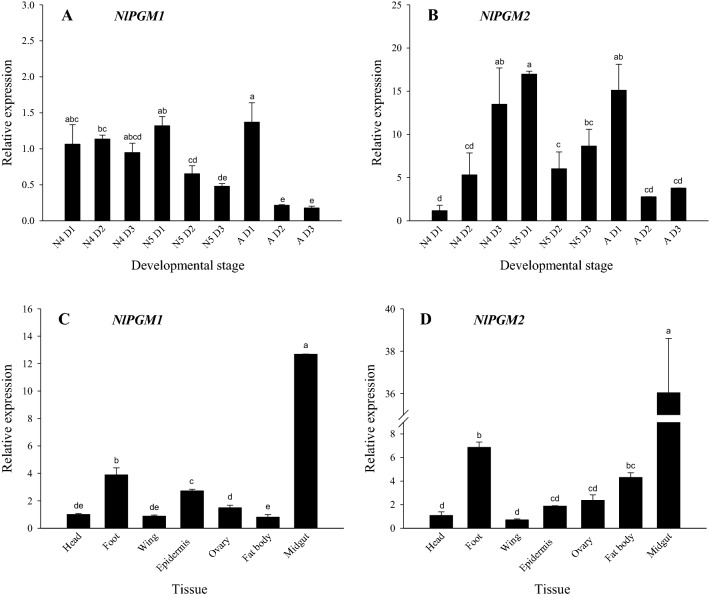
Relative expression of *PGM1* and *PGM2* in different tissues and developmental stages in the brown planthopper. Expression patterns of *PGM1* (**a**) and *PGM2* (**b**) at different developmental stages from the first day of the fourth instar nymph to the third day of adulthood. The expression patterns of *PGM1* (**c**) and *PGM2* (**d**) in various tissues including head, foot, wing, epidermis, ovary, and fat body collected from adults. The mRNA levels were normalised to *N. lugens* 18S (*Nl18S*) mRNA, and the relative expression levels for each tissue and developmental stage were measured in relation to the expression levels obtained in the head of the insects and first day of the fourth instar nymph. One-way analysis of variance (ANOVA) was performed to test for statistical significance. Data are presented as the mean ± SD (*N* = 3). Means with different letters within the same group differ significantly, *P* < 0.05

First, we examined the expression levels of the *PGM1* and *PGM2* genes at different developmental stages, from the first day of the fourth instar to the third day of adulthood. At the fourth instar nymph stage, *PGM1* and *PGM2* had different expression patterns. The expression level of *PGM1* was relatively stable, while the expression level of *PGM2* in the fourth instar nymph increased gradually. The expression levels of *PGM1* and *PGM2* were high at the initial stage of the fifth instar nymph, and then decreased. At the adult stage, the expression levels of the *PGM1* gene were the highest on the first day of adulthood and then decreased significantly and remained at relatively low levels on the second and third days (Fig. 1a, b).

Both *PGM1* and *PGM2* were most highly expressed in the midgut, followed by the foot. The expression level of the *PGM1* in the epidermis was lower than in the foot; the expression levels in the head, wings, ovary, and fat body were relatively low overall. The expression level of *PGM2* in the fat body was lower than in the foot; the expression levels in the head, foot, wing, and ovary were also relatively low overall (Fig. 1c, d).

### Evaluation of the efficiency of RNAi knockdown by dsPGM1 and dsPGM2

**Fig. 2:**
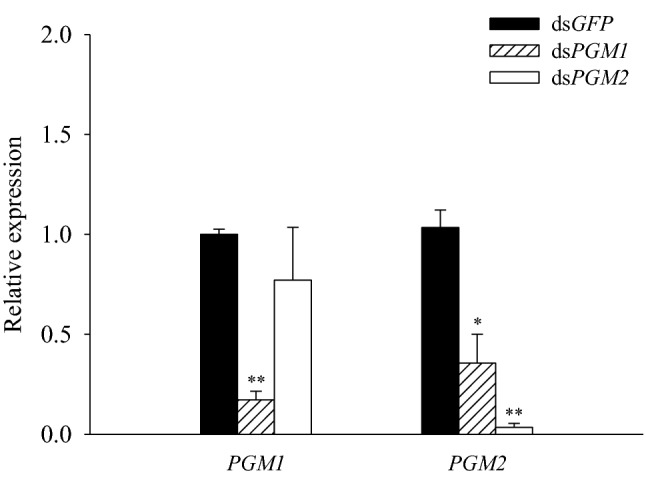
Relative expression levels of *PGM1* and *PGM2* after RNAi. mRNA levels of *NlPGM1* and *NlPGM2* relative to the *Nl18S* RNA level following RNAi targeting. The first day of the fifth instar nymph was chosen for dsRNA injection. *Indicates a significant difference and ** indicates an extremely significant difference

The expression levels of *PGM1* and *PGM2* were significantly decreased after RNAi for 48 h. Knocking down the *PGM1* gene, the relative expression of PGM1 decreased almost 5 times compared to the control group (Fig. 2). In addition, the relative expression of PGM2 in the dsPGM2 group was 33 times lower than that in dsGFP group (Fig. 2). Moreover, compared with the control group injected with dsGFP, the expression levels of the *PGM2* gene decreased slightly after the *PGM1* knockdown, but this decrease was not significant. The expression level of the *PGM1* gene also decreased significantly after the *PGM2* knock down (Fig. 2).

### Expression of trehalose and glycogen metabolism-related genes following PGM1 or PGM2 knockdown

**Fig. 3:**
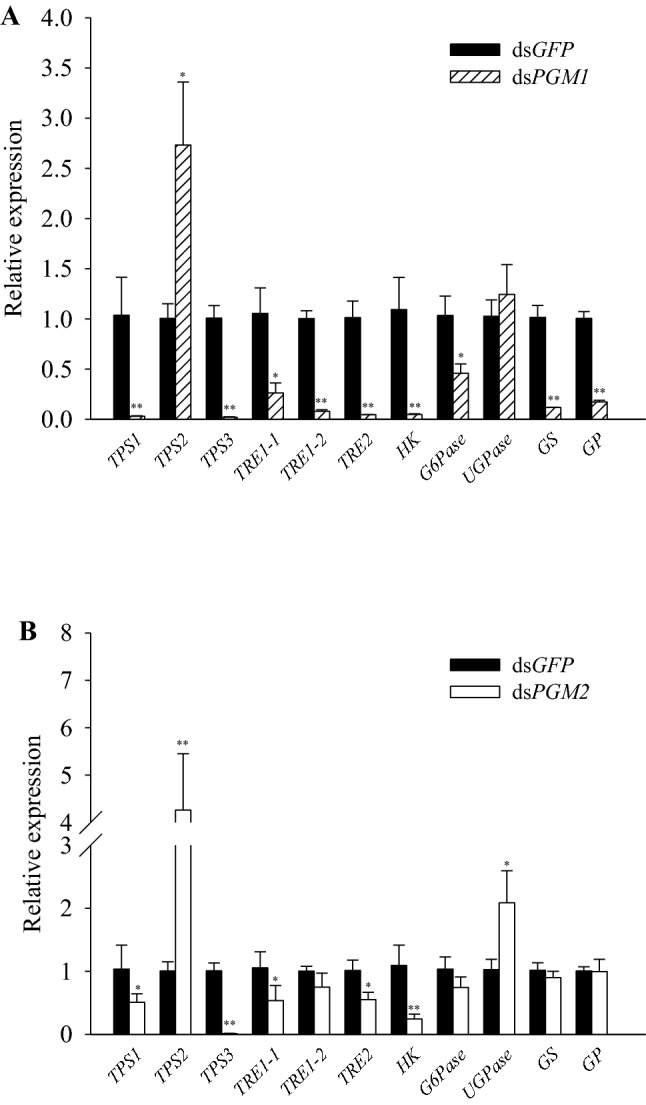
Expression levels of trehalose and glycogen metabolic pathway genes after RNAi. The expression levels of three trehalose-6-phosphate synthases (TPSs), three trehalases (TREs), hexokinase (HK), glucose-6-phosphatase (G6Pase), UDP-glucose pyrophosphorylase (UGPase), glycogen synthase (GS), and glycogen phosphorylase (GP) at 48 h relative to the *N. lugens* 18S (*Nl18S*) RNA level following PGM1 (**a**) or PGM2 (**b**) knockdown

The expression of most genes related to trehalose and glycogen metabolism were significantly decreased after knockdown of the *PGM1* gene for 48 h, with the exception of the expression level of *trehalose-6-phosphate synthase 2* (*TPS2*)*,* which significantly increased, and the expression level of *UDP-glucose pyrophosphorylase* (*UGPase*), which showed no significant change (Fig. 3a). In contrast, the expression levels of *TPS1*, *TPS3*, *trehalase 2* (*TRE2*)*,* and *hexokinase* (*HK*) were significantly decreased after knockdown of *PGM2* for 48 h. The expression levels of *TPS2* and *UGPase* increased significantly, while the remaining genes showed no significant changes (Fig. 3b).

### Effects of *PGM1* and *PGM2* knockdown on glycogen, trehalose, and glucose levels

**Fig. 4:**
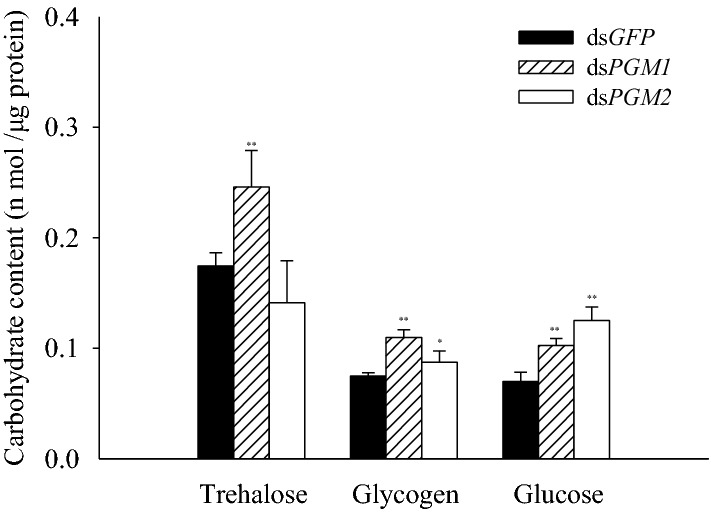
Trehalose, glycogen, and glucose content of *N. lugens* after RNAi. All measurements were performed in triplicate

Compared with the control group, both the glycogen and glucose contents showed significant increases after *PGM1* or *PGM2* knock down for 48 h. In addition, knockdown of *PGM1*, but not of *PGM2*, resulted in a significant increase in the trehalose content (Fig. 4).

### Effects of *PGM1* and *PGM2* knockdown on trehalase activity

**Fig. 5:**
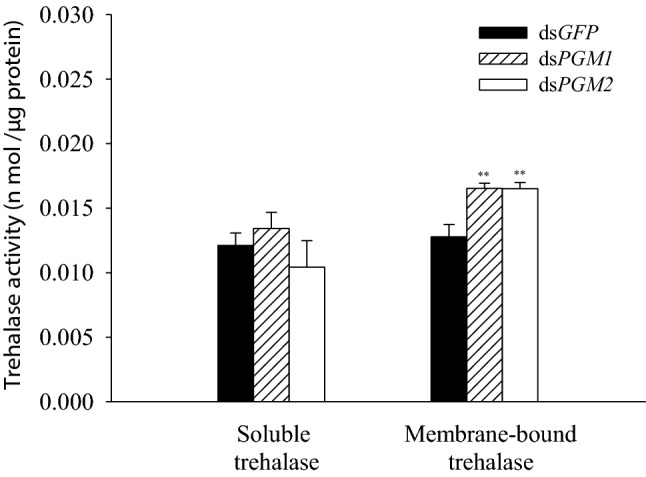
Trehalase 1 (soluble trehalase, a) and trehalase 2 (membrane-bound trehalase, b) activities after RNAi. All measurements were performed in triplicate

Compared with the dsGFP group, knockdown of *PGM1* or *PGM2* caused a significant increase in membrane-bound, insoluble, trehalase activity (Fig. 5).

### Expression of chitin metabolism-related genes after *PGM1* and *PGM2* gene knockdown

**Fig. 6:**
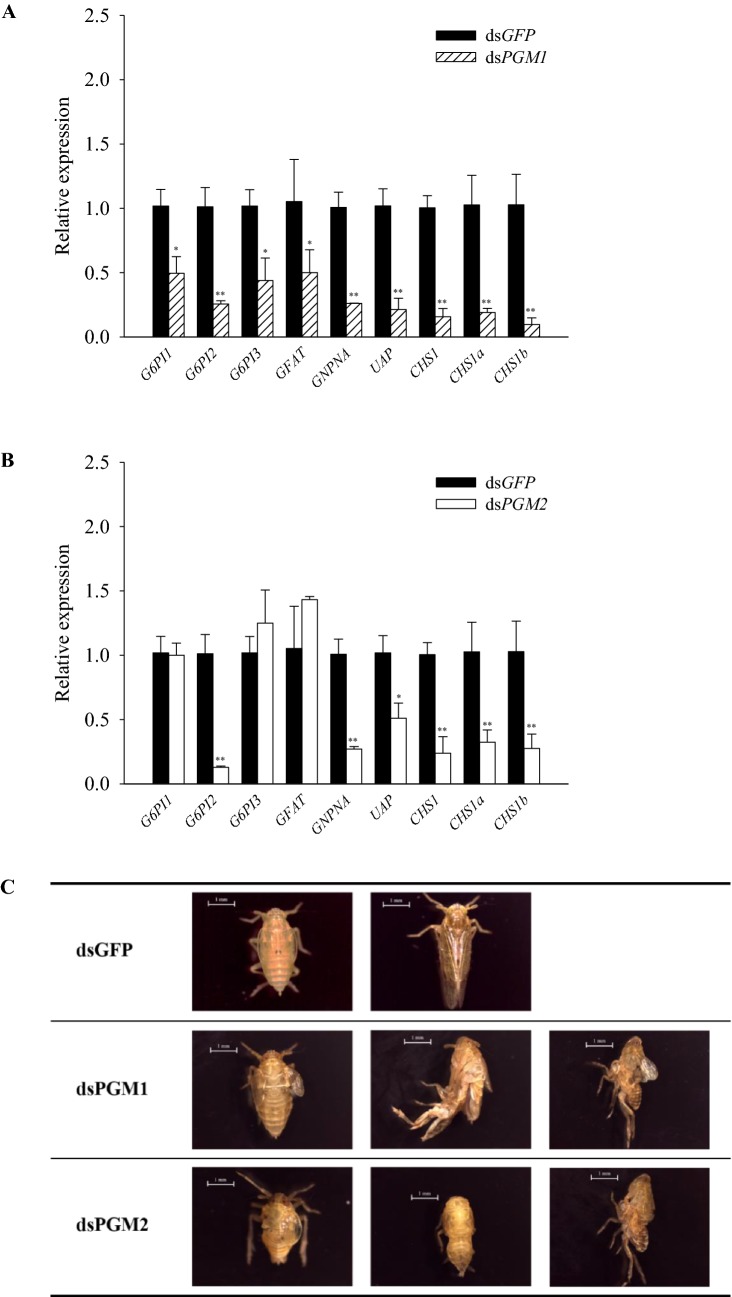
Expression levels of genes in the chitin metabolic pathway and the resulting abnormal phenotype after RNAi. **a**, **b** Expression levels of three glucose-6-phosphate isomerases (G6PIs), glutamine: fructose-6-phosphate aminotransferase (GFAT), glucosamine-6-phosphate *N*-acetyltransferase (GNPNA), UDP-*N*-acetylglucosamine pyrophosphorylase (UAP), and three chitin synthases (CHSs) relative to the *N. lugens* 18S (*Nl*-*18S*) RNA level. **c** Abnormal insects at the larva-adult stage

Compared with the control group injected with dsGFP, the expression levels of all studied genes related to chitin metabolism were significantly decreased (1–9 times) after the *PGM1* knockdown (Fig. 6a). The *PGM2* knockdown also significantly decreased the expression of most of the genes involved in chitin metabolism, except for *G6PI1* and *G6PI3,* which were not significantly changed, and *glutamine:fructose-6-phosphate aminotransferase* (*GFAT*), which was significantly increased (Fig. 6b). Knockdown of *PGM1* and *PGM2* also caused clear wing deformities and varying degrees of difficulty in molting (Fig. 6c).

## Discussion

According to sequence analysis, PGM1 and PGM2 are highly conserved among different insects, and they are also highly conserved during evolution (Figs. S2, S3). As we all known, PGM catalyses the interconversion of (G-1-P) and (G-6-P). G-1-P is an important intermediate in the metabolism of glycogen, galactose, glycoproteins, and glycolipids; therefore, PGM is involved in a variety of cellular functions (Hakomori 1985; Gahmberg and Hakomori 1973; Novelli and Reichardt 2000). G-6-P is a central, metabolite, part of glycolysis and the pentose phosphate pathway, providing precursors for anabolic pathways and cofactors required for cell proliferation (Ward and Thompson 2012). PGM is widely found in animals, plants, and microorganisms and is distributed in almost all tissues (Egli et al. 2010; Stray-Pedersen et al. 2014; Weyler and Heinzle 2015). In the brown planthopper, both *PGM1* and *PGM2* were most highly expressed in the midgut, followed by the foot (Fig. 1c, d). While the role of PGM in the intestine is likely related to nutrient intake and utilisation and heterogeneous metabolism (insecticide metabolism) (Bao et al. 2012), we speculate that *PGM* may also play an important role in substance metabolism in the brown planthopper. Given that the *PGM1* and *PGM2* genes are highly expressed on the first day of the fifth instar and on the first day of the adult (Fig. 1a, b), PGM1 and PGM2 may play an important role in chitin synthesis.

RNAi technology has become an effective tool in the research of insect gene function, gene expression regulation, pest control, and new pesticide development (Lou et al. 2018; Han et al. 2018). Although the brown planthopper is an agricultural pest, it is also a desirable model insect to study gene function (Xi et al. 2015a, b). We used RNAi technology to explore the role of *PGM* in regulating the metabolism of trehalose and glycogen in the brown planthopper. To verify the specificity and effectiveness of dsPGM1 and dsPGM2 for RNAi, we first examined the relative expression levels of *PGM1* and *PGM2* after injection with dsPGM1 and dsPGM2, respectively. We found that the relative expression levels of *PGM1* and *PGM2* were significantly decreased after RNAi injection (*P* < 0.01). In addition, the *PGM1* levels were relatively unaffected after *PGM2* knockdown, whereas the *PGM2* expression levels were significantly decreased after *PGM1* knockdown (Fig. 2). This suggests that the expression level of *PGM1* may affect the expression level of *PGM2*.

Trehalose is the blood sugar in insects and critical for insect growth, development, and molting (Elbein 1974; Elbein et al. 2003; Tang et al. 2014). Glycogen is an important energy store. The expression levels of the genes involved in trehalose and glycogen metabolism were significantly down-regulated after the *PGM1* knocking down (Fig. 3a). Although some genes were unaffected by the knockdown of *PGM2*, others showed a slight down-regulation (Fig. 3b). The trehalose content was significantly increased after the dsPGM1 injection, but no significant effect was observed after the dsPGM2 injection (Fig. 4). This is consistent with the extremely low expression levels of the *TRE* genes and a significant increase of expression in the *TPS2* gene following the dsPGM1 injection (Fig. 3). Previous studies have shown that when different types of *TRE* genes are knocked down, the changes in trehalose content are different (Yang et al. 2017; Chen et al. 2010), which may explain why the trehalose content differs after injection of dsPGM1 compared to dsPGM2.

The glucose levels were also significantly increased after injection of both dsPGM1 and dsPGM2 (Fig. 4). This is consistent with the several-fold higher expression level of *G6Pase* compared to the expression level of *HK*, even though *HK* and *G6Pase* were both suppressed by dsPGM1 and dsPGM2 (Fig. 3). Studies have shown that knockdown of the *HR38* gene in *Aedes aegypti* can block transcriptional activation of the *CM* gene encoding PGM and TPS, resulting in increased glycogen accumulation (Dong et al. 2018). Here, we also found that the levels of glycogen in the brown planthopper were significantly increased after injection of dsPGM1 and dsPGM2 (Fig. 4). Trehalose and trehalase activity are key for the regulation of various physiological processes in insects (Ge et al. 2011). Membrane-bound trehalase activity was significantly increased after dsPGM1 or dsPGM2 injection, whereas soluble trehalase activity was not significantly altered (Fig. 5). These results indicate that *PGM1* and *PGM2* may have greater effect on membrane-bound trehalase than on the soluble form of the enzyme.

An increasing number of studies have shown that trehalose metabolism can regulate the synthesis of chitin through trehalose synthase and trehalose hydrolase (Tang et al. 2010; Shukla et al. 2015; Shi et al. 2016; Yang et al. 2017). Knockdown of the *TRE* gene in the brown planthopper caused clear deformities in the wings and difficulty in molting, indicating that trehalose has a direct effect on chitin synthesis (Zhang et al. 2017a). Abnormal synthesis of chitin has also been observed in the brown planthopper after knockdown of the *TPS* gene (Yang et al. 2017). In this study, both the *TPS* and *TRE* genes were affected to some degree after knockdown of the *PGM1* or *PGM2* genes; therefore, we further tested the effect of *PGM1* or *PGM2* knockdowns on chitin synthesis. The expression of most of the genes involved in chitin synthesis were significantly decreased after ds*PGM1* or dsPGM2 injection (Fig. 6a, b), and there were different degrees of deformity (Fig. 6c), suggesting that silencing the *PGM1* or *PGM2* genes can inhibit the expression of chitin synthase (*CHS*), resulting in the inability of the brown planthopper to synthesise chitin. Recently, in a study in the *A. pisum* nymph, knockdown of *A. pisum CHS* was found to disrupt embryonic development, leading to difficulty in molting (Ye et al. 2019). Another study in whitebacked planthoppers showed malformations and high mortality after *CHS1*, *CHS1a*, and *CHS1b* knockdown (Wang et al. 2019). These studies demonstrate that CHS plays a pivotal role in chitin synthesis, which is consistent with our results.

## Conclusions

The expression patterns of the *PGM1* and *PGM2* genes were similar, both at different developmental stages and in different tissues. RNAi using ds*PGM1* and ds*PGM2* effectively inhibited the expression of their respective target genes and disrupted the normal metabolic balance between glycogen and trehalose, which affected chitin metabolism.

## Conflict of interest

The authors declare that the research was conducted in the absence of any commercial or financial relationships that could be construed as a potential conflict of interest.

## Electronic supplementary material

Below is the link to the electronic supplementary material.

Supplementary file1 (DOCX 331 kb)
