# Supplementary material for: Role of phosphoglucomutase in regulating trehalose metabolism in Nilaparvata lugens
Source: 3 Biotech. 2020 Jan 23;10(2):61. doi: 10.1007/s13205-020-2053-5 (PMC6977789; doi:10.1007/s13205-020-2053-5)
Supplement: Supplementary file 1 — Supplementary file1 (DOCX 331 kb) [file 13205_2020_2053_MOESM1_ESM.docx]

**
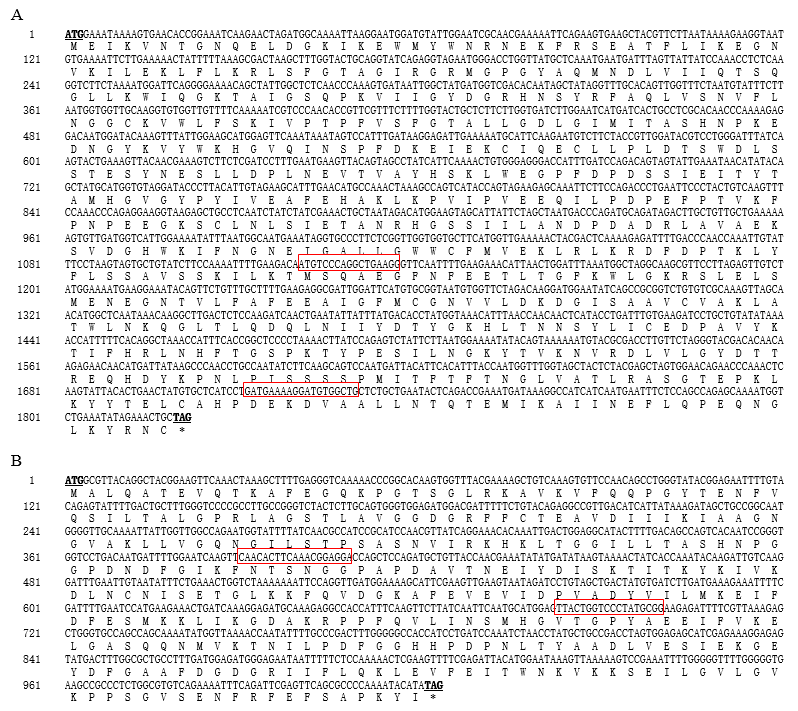
**

**
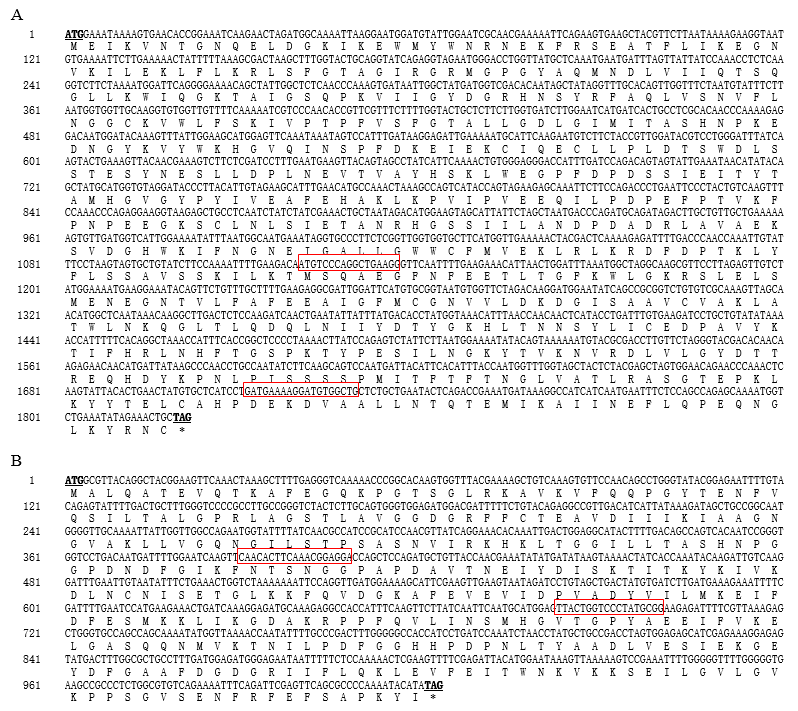
**

**Figure S1 Analysis of nucleotide and deduced amino acid sequences of *NlPGM1* and *NlPGM2*.** Initiation and termination codons are indicated in bold and underlined font; the termination codon before the first Met is also indicated in bold and underlined. The target regions of dsPGM1 and dsPGM2 are framed in red. The nucleotide sequences reported in this paper have been submitted to GenBank (accession numbers: KU556839.1 and KU556840.1). A: NlPGM1; B: NlPGM2.

**A**

**B**

**Figure S2 Alignment of PGM1 and PGM2 Amino acid sequences from different insect species.** A: Alignment of NlPgm1 (*Nilaparvata lugens*, AQT25654.1), LsPgm2-like (*Laodelphax striatellus*, RZF37013.1), ObbPgm2 (*Osmia bicornis bicornis*, XP_029048774.1), SiPgm2 (*Solenopsis invicta*, XP_011164321.1), ClPgm2 (*Cimex lectularius*, XP_014251917.1), HhPgm2 (*Halyomorpha halys*, XP_014286651.1), MpPgm2 (*Monomorium pharaonis*, XP_012529987.1), and CcPgm2 (Ceratina calcarata, XP_017879794.2) using DNAMAN software. B: Alignment of NlPgm2 (*Nilaparvata lugens*, AQT25655.1), LsPgm1 (*Laodelphax striatellus*, RZF40858.1), FoPgm1 (*Frankliniella occidentalis*, XP_026292338.1), LmPgm1 (*Locusta migratoria*, ACM78949.1), MsPgm1 (*Melanaphis sacchari*, XP_025208423.1), SfPgm1 (*Sipha flava*, XP_025415472.1), ApPgm1 (*Acyrthosiphon pisum*, XP_008182079.1), and MpPgm1 (*Myzus persicae*, XP_022164622.1) using DNAMAN software. Highly conserved regions are shown in yellow and blue.

**
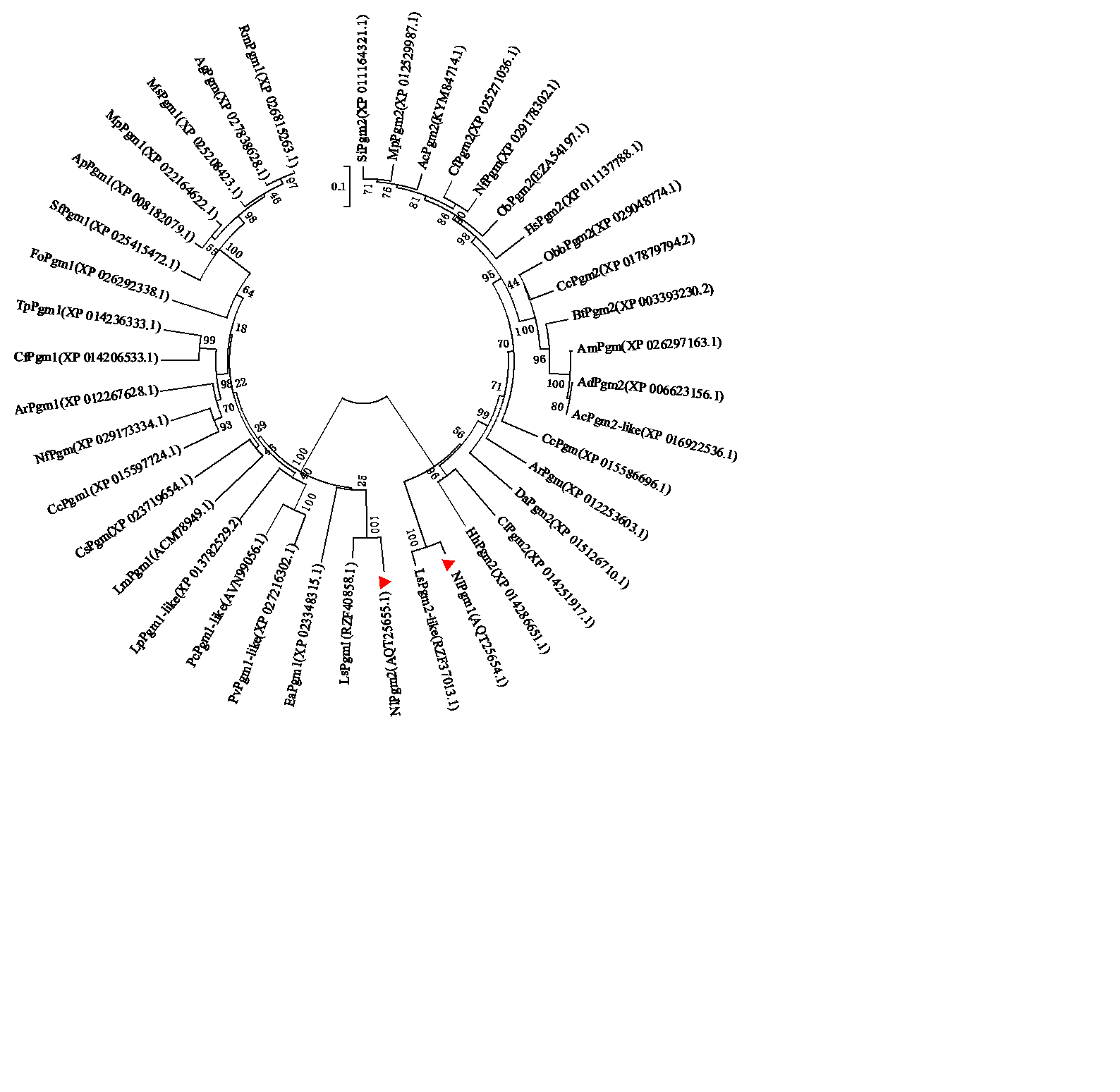
**

**Figure S3 Phylogenetic tree constructed using the amino acid sequences of some known insects’ phosphoglucomutase.** Full-length amino acid sequences were aligned using the Mega 6.0 software. A bootstrap analysis was carried out and the robustness of each cluster was verified using 1000 replicates. Values at the cluster branches indicate the results of the bootstrap analysis. The phosphoglucomutase family proteins were NlPgm1(*N. lugens*), LsPgm2-like (*L. striatellus*), ObbPgm2 (*O. bicornis bicornis*), SiPgm2 (*S. invicta*), ClPgm2 (*C. lectularius*), HhPgm2 (*H. halys*), MpPgm2 (*M. pharaonis*), CcPgm2 (*C. calcarata*), CfPgm2 (*C. floridanus*), AdPgm2 (*A. dorsata*), AmPgm (*A. mellifera*), ObPgm2 (*O. biroi*), HsPgm2 (*H. saltator*), NfPgm (*N. fulva*), ArPgm (*A. rosae*), CcPgm (*C. cinctus*), AcPgm2-like (*A. cerana*), AcPgm2 (*A. colombica*), DaPgm2 (*D. alloeum*), BtPgm2 (*B. terrestris*), NlPgm2 (*N. lugens*), LsPgm1 (*L. striatellus*), FoPgm1 (*F. occidentalis*), LmPgm1 (*L. migratoria*), MsPgm1 (*M. sacchari*), SfPgm1 (*S. flava*), ApPgm1 (*A. pisum*), MpPgm1 (*M. persicae*), PvPgm1-like (*P. vannamei*), AgPgm (*A. gossypii*), RmPgm1 (*R. maidis*), CfPgm1(*C. floridanum*), PcPgm1-like (*P. clarkii*), CcPgm1 (*C. cinctus*), CsPgm (*C. secundus*), NfPgm (*N. fulva*), EaPgm1 (*E. affinis*), ArPgm1 (*A. rosae*), LpPgm1-like (*L. polyphemus*), and TpPgm1 (*T. pretiosum*). Characters in parentheses are the GenBank accession numbers. The triangles in red indicate the phosphoglucomutase amino acid sequences of brown planthopper.
